# Perspective: Engineering noise in biological systems towards predictive stochastic design

**DOI:** 10.1063/1.5025033

**Published:** 2018-05-07

**Authors:** Roy D. Dar, Ron Weiss

**Affiliations:** 1 Department of Bioengineering, University of Illinois at Urbana-Champaign, Urbana, Illinois 61801, USA; 2 Carl R. Woese Institute for Genomic Biology, University of Illinois at Urbana-Champaign, Urbana, Illinois 61801, USA; 3 Center for Biophysics and Quantitative Biology, University of Illinois at Urbana-Champaign, Urbana, Illinois 61801, USA; 4 Department of Biological Engineering, Massachusetts Institute of Technology, Cambridge, Massachusetts 02139, USA; 5 Department of Electrical Engineering and Computer Science, Massachusetts Institute of Technology, Cambridge, Massachusetts 02139, USA

## Abstract

Significant progress has been made towards engineering both single-cell and multi-cellular systems through a combination of synthetic and systems biology, nanobiotechnology, pharmaceutical science, and computational approaches. However, our ability to engineer systems that begin to approach the complexity of natural pathways is severely limited by important challenges, e.g. due to noise, or the fluctuations in gene expression and molecular species at multiple scales (e.g. both intra- and inter-cellular fluctuations). This barrier to engineering requires that biological noise be recognized as a design element with fundamentals that can be actively controlled. Here we highlight studies of an emerging discipline that collectively strives to engineer noise towards predictive stochastic design using interdisciplinary approaches at multiple-scales in diverse living systems.

Fluctuations in gene expression, cell-to-cell signaling, and cell environment are intrinsic to the blueprints of life. Such fluctuations (or “noise”) are not necessarily detrimental to function, and nature has evolved genome-wide mechanisms for both suppressing and exploiting it. Gene expression noise of genes vital to cell function and development is tightly regulated and often attenuated. For example, the *Escherichia coli* transcription factor network^1^ and stem cell pluripotent factors^2^ are enriched with negative autoregulatory loops known to increase robustness and suppress noise.^3^ Negative autoregulation also shifts noise to higher frequencies to ease noise filtering by downstream signal transduction and regulatory cascades.^4^ In addition, organisms have evolved regulatory architectures for responding to fluctuating environments,^5,6^ and natural stochastic design of network modules may evolve under specific selection pressures.^7^ Noise can be enhanced and exploited for improved cellular response and increased fitness advantage. Stress response genes facing uncertain and fluctuating environments have promoter regulation that enhances noise,^8^ such as the TATA box (a DNA sequence important for transcription found in the core promotor region of genes in archaea and eukaryotes) or high nucleosome occupancy.^6,9^ Exploitation of noise occurs in a variety of species and at multiple-scales including decision-making of lambda-phage^10^ and human immunodeficiency virus (HIV)^11^ and *Bacillus subtilis* competence^12^ and in drug resistance of bacteria^13^ and cancer.^14^ Understanding the fundamentals of nature's processing of biological fluctuations will provide principles to forward-design synthetic tuning and modulation of noise in living systems, enabling advancements in synthetic biology, tissue engineering, therapeutics, nanobiotechnology, and more (Fig. 1).

**FIG. 1.**
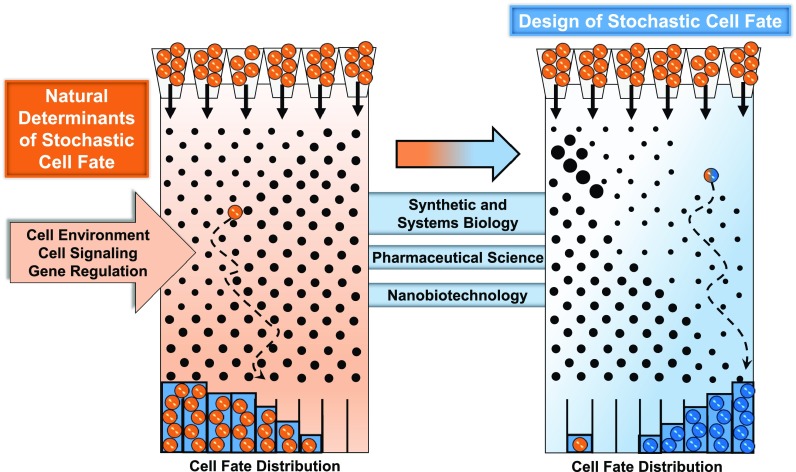
Stochastic design of natural determinants of cell fate. (Left) Natural determinants of stochastic cell fate are depicted as an ordered pattern of nails on a board. Individual orange cells fall semi-randomly into a biased and regulated probability distribution determined by the cell environment, cell signaling, and gene regulation. In this example, natural determinants and regulation would define the nail composition, size, and patterning. (Right) Synthetic and systems biology, pharmaceutical science, and nanobiotechnology are a subset of approaches for stochastic design to actively bias blue cells into a new fate distribution by modifying nail patterns, sizes, and compositions.

Bioengineers commonly face concepts of “heterogeneity,” “variability,” “error,” and “noise” in their research. Similar to error bars, natural variability and biological fluctuations are perceived with some intrinsic rigidity, or as inevitable, outside of our control. However, recent studies provide strategies to actively modulate noise independent of mean expression levels. Inducible synthetic gene circuits consisting of negative autoregulation,^15^ arrangements of two transcriptional regulators,^16^ mutated TATA boxes,^17,18^ and variable repressor binding site locations^19^ have been constructed for precise noise modulation. Additionally, noise drug screening for changes in variability, but not mean protein levels, has uncovered an orthogonal axis for drug discovery and synergies.^20^ Treatments with noise enhancer and suppressor compounds were demonstrated to bias HIV decision-making in populations of latently infected cells. Advancing our ability to actively tune noise in gene expression and multi-cellular heterogeneity will open a new toolbox for predictive stochastic design of biology and complement the diversity of synthetic approaches currently aimed at regulating average gene expression levels but not the variability of a targeted molecular species. Predictive stochastic design will enable the shaping of phenotypic distributions and statistical attributes of complex cellular systems, devices, and applications.

The future of bioengineering will require the development of a new framework for *engineering noise* in diverse systems. Heterogeneity and gene expression noise need to no longer be looked at as a simple byproduct of living systems but as an essential component in system design. A formal discipline addressing this void for understanding, quantifying, communicating, and engineering biological noise has yet to be integrated within interdisciplinary research communities. Research efforts to engineer and modulate stochasticity provide new perspectives and tools within diverse fields ranging from synthetic^17,19^ and systems biology,^21,22^ multicellular tissues,^23,24^ nanobiotechnology,^25^ drug discovery,^20^ and disease.^26^ Noise engineering in these contexts shows promise towards the control of tissue patterning for human health and curing disease or in engineered plants for global food security, the environment, and bioenergy.

Previously, Lu *et al.* reported a computational study of a “noise generator.” The authors demonstrated that tuning noise across a defined noise phase space can modulate the dynamics of a positively auto-regulated gene circuit between unimodal and bimodal regimes of gene expression.^27^ The study suggests that future noise tuning may actively modulate diverse gene circuits, motifs, and networks for controlling dynamic cellular processes, states, and decision-making. Recent investigations have shown that activators and chromatin-modifying compounds [e.g., protein kinase C (PKC) agonists, Histone deacetylase inhibitors (HDACis), and DNA methyltransferase (DNMT) inhibitors] modulate gene expression noise.^20,28^ Building on these studies, in this issue, Megaridis *et al.* demonstrate fine-tuning of noise in epigenetic regulation by controlled inhibition of nucleosome remodeling in the HIV-1 promoter.^22^ The authors apply a combination of drug treatments to map an extended noise phase space by modulating different sources of noise at variable strengths. They demonstrate consistent noise tuning at multiple integration sites, suggesting its application across the genome for future integration of advanced gene circuits. Despite inhibiting a global nucleosome remodeling complex with treatments [including a Food and Drug Administration (FDA) approved drug], the authors observe consistent and robust fine-tuning of noise intrinsic to gene expression from the HIV promoter. This suggests that nucleosome remodeling inhibitors can provide targeted regulation for specific tuning of noise of the HIV and potentially other promoters when used at low concentrations to avoid cell death. This also demonstrates that promoter-specific noise can be finely tuned despite the broad-acting inhibition of nucleosome remodeling and its off-target effects on other promoters. Fine-tuning promoter noise with drug treatments demonstrates exogenous and time-dependent control of noise of cell populations without the integration and design of synthetic gene circuits. Collectively, the combination of computation, exogenous drug treatments, and synthetic biology provide opportunities for multi-modal design of noise.

In addition to single-cells, multi-scale noise tuning of intracellular gene expression, cell-to-cell signaling, and tissue microenvironment may advance our understanding of heterogeneity in multi-cellular systems. Miller *et al.* reported a modular design approach for synthesis of artificial tissue and control of its heterogeneity.^23^ Computational and theoretical analyses of synthetic gene modules for generating population level diversity created a system with increased robustness to uncertain environments. Interestingly, features often associated with reduced system robustness, such as multicellular asynchrony and noise amplification, were found to be beneficial for tissue homeostasis. Engineering of epigenetics^20,22^ and *cis* regulation^17,19^ at the promoter level along with synthetic design of cellular heterogeneity with gene circuit modules^23^ provide the initial steps towards establishing multi-scale and spatiotemporal noise engineering of cellular systems in dynamic environments. Collectively, the combination and development of novel multi-scale approaches will contribute to the success of engineering noise and further guide the control of heterogeneity in multi-cellular systems.

As a fundamental engineering component, understanding and tuning noise, variability, and heterogeneity will enhance our research and bioengineering capabilities. Similar to other top-down and bottom-up approaches, engineering noise will facilitate a deeper understanding of systems biology and structure-function relationships of noise in natural complex systems. To enable this change, peer-reviewed journals would benefit from soliciting focus issues solely dedicated to noise control and stochastic design to a highly interdisciplinary research community. In addition, the research community may benefit from holding joint meetings focused on *engineering noise in living systems* which bring together biophysicists, systems and synthetic biologists, and tissue and bioengineers. Advancing the fundamentals to engineer noise will unpeel layers from the complexity faced with bioengineering life.
